# First Report of *Proteocephalus longicollis* (Zeder, 1800) in *Alosa fallax lacustris* (Fatio, 1890) from Lake Garda (Italy): Morphological and Molecular Study

**DOI:** 10.3390/vetsci10090567

**Published:** 2023-09-11

**Authors:** Ivan Corti, Giovanni De Benedetto, Kristian Riolo, Renato Malandra, Gabriella Gaglio

**Affiliations:** 1 Agenzia di Tutela Della Salute Dell’insubria, 22100 Como, Italy; corti.ivan@gmail.com; 2 Department of Veterinary Sciences, University of Messina, 98168 Messina, Italy; gdebenedetto@unime.it; 3 Department of Chemical, Biological, Pharmaceutical and Environmental Sciences, University of Messina, 98166 Messina, Italy; kristian.riolo@unime.it; 4 Veterinario Già Responsabile S.S. Mercati Generali, ATS Città Metropolitana di Milano, 20137 Milano, Italy; renato.malandra55@gmail.com

**Keywords:** tapeworm, Proteocephalidae, twaite shad, freshwater fish, northern Italy

## Abstract

**Simple Summary:**

Twaite shad (*Alosa fallax*) includes two subspecies, *Alosa fallax nilotica* and *Alosa fallax lacustris*, representing the only landlocked Clupeidae inhabiting Italian deep-water lakes. Studies on the parasitic fauna of this species in Italy are, to date, limited. The aim of the present study was to evaluate the presence of tapeworms from *A. fallax lacustris* sampled in Lake Garda. Sixty-six *A. fallax lacustris* specimens were evaluated during official veterinarian checks at the Milan fish market (Italy) to detect the presence of helminths. All parasites found were fixed in 70% ethanol and transferred to the laboratories of the University of Messina for morphological and molecular analysis. Some parasites were stained with Semichon’s carmine red technique for morphological evaluation, while molecular analysis was carried out on six replicates using LSU rRNA and ITS2 region genes. Eight specimens out of sixty-six were positive for adult tapeworms within the pyloric caeca. Analysis could to identify the parasites as *Proteocephalus longicollis*, both morphologically and molecularly. This species, not considered a zoonotic agent, represents a low risk of parasitic fish-borne zoonosis for consumers of *A. fallax lacustris* from Lake Garda.

**Abstract:**

Twaite shad (*Alosa fallax*) includes two subspecies, the anadromous *Alosa fallax nilotica*, and the landlocked species *Alosa fallax lacustris*, representing the only Clupeidae inhabiting Lake Garda. Study of the parasitic fauna of this species in this area is still limited. For this reason, the aim of the present study was to evaluate the presence of tapeworms from *A. fallax lacustris* sampled in Lake Garda. Sixty-six *A. fallax lacustris* specimens were collected at the Milan fish market (Lombardy, Italy); specifically, an evaluation of the gastrointestinal contents was carried out for the presence of helminths. All parasites found were fixed in 70% ethanol and transferred to the laboratories of the University of Messina for morphological and molecular analysis. Parts of the parasites were stained with Semichon’s carmine red technique. Molecular analysis was carried out using LSU rRNA and ITS2 region genes. Eight specimens out of sixty-six (12.1%) were positive for adult cestodes within the pyloric caeca. Morphological and molecular analysis could to identify the parasites found to be *Proteocephalus longicollis*. This parasite species is not considered a zoonotic agent, representing a low risk of parasitic fish-borne zoonosis for consumers of this appreciated fish from Lake Garda.

## 1. Introduction

Twaite shad, *Alosa fallax* (Lacépède, 1803), includes two subspecies present in the Mediterranean area. Among these, *Alosa fallax nilotica* is an anadromous species characterized by migratory behavior: this species usually moves between seawater and freshwater, mainly for reproduction activities, while *Alosa fallax lacustris* (Fatio, 1890) is a landlocked species, reported to be the only teleost belonging to the Clupeidae family present in Italian deep lakes, such as Lake Garda [1]. Since 1916, some hypotheses regarding the differentiation of this subspecies have been presented [2]. Total adaptation to freshwater during all the development stages, including reproduction, has been reported, meaning that *A. fallax lacustris* is considered an adapted landlocked species [2]. In the Mediterranean area, few *A. fallax lacustris* populations have been recognized, mainly in northern and central Italy [3]. Moreover, according to Baglinière [3], few data on *A. fallax lacustris* distribution are present for the Mediterranean area. Other authors suggested that the *A. fallax lacustris* populations present in Italian lakes were isolated by tectonic changes, as reported by Aprahamian et al. [4]. Moreover, considering the report from Trewavas [5], *A. fallax lacustris* represents the only shad present in Europe, confined to Italian lakes such as Garda, Como and Bracciano, where this teleost can develop and pass its entire life cycle without moving between freshwater and seawater, even if, as reported by Taddei et al. [6], the inland population has disappeared from some Italian lakes, such as Vico and Bolsena. From the morphological point of view, some differences between *A. fallax lacustris* and *A. fallax nilotica* have been described [7]. As reported by Tortonese [8], the main morphological distinction is the wider body and longer head characteristic of *A. fallax nilotica* in comparison to *A. fallax lacustris.* According to Quignard and Douchement [9], the most important morphological characteristics used to assess the correct identification between the subspecies is the variability of the gill raker number, which is 35–45 in *A. fallax nilotica*, while the *A. fallax lacustris* has 50–60 gill rakers. These data were previously reported also by Ivanovic [10]. In Italy, *A. fallax lacustris* can reach a total length (TL) and body weight (BW) of 38 cm and 500 g, respectively [11,12]. The *A. fallax lacustris* diet is mainly composed of planktonic organisms. In populations of northern Italian lakes, the diet is composed mainly of crustaceans belonging to the Branchiopoda and Copepoda classes and is strongly influenced by seasonal variations. According to the seasons, copepods are the most important nutrient in winter and autumn, while branchiopods are the main components in spring and summer [11,13]. Data regarding the *A. fallax lacustris* Italian population reported that, in summer, the diet is composed of *Daphnia hyalina* (Leydig, 1860), while in the other seasons this species prefers copepods [14]. In Italy, few studies have been performed to investigate the parasite fauna of *A. fallax lacustris*. Among them, the presence of *Diphyllobothrium latum* (Linnaeus, 1758) has been studied by Gustinelli and colleagues [15], reporting no positivity for plerocercoid larvae in *A. fallax lacustris* from four big lakes in the north of Italy. According to Menconi et al. [16], the presence of the nematode *Eustrongylides excisus* (Ägerskiöld, 1909) has been investigated in *A. fallax lacustris* from Lake Garda, with only negative results. In Europe, the genus *Alosa* has been investigated for parasites, considering mainly anadromous and marine species such as *Alosa alosa* (Linnaeus, 1758) and *A. fallax nilotica;* a study carried out by Bao et al. [17] reported the presence of *Anisakis simplex* (Rudolphi, 1809) and *Anisakis pegreffii* (Campana-Rouget & Biocca, 1955) in both *A. alosa* and *A. fallax lacustris* from the Western Iberian Peninsula Rivers. Rokicki and colleagues [18] reported the presence of metacercariae belonging to the genus *Diplostomum*, as well as nematode larvae (L3) identified as *Contracaecum osculatum* (Rudolphi, 1802) *Desmidocercella numidica* (Seurat, 1920) and *Hysterothylacium aduncum* (Rudolphi, 1802) from Twaite shad caught in the southern Baltic Sea.

Tapeworms belonging to the genus *Proteocephalus* (Weinland, 1858) are usually present in freshwater teleosts, mainly from the Palaearctic areas [19]. Crustaceans characterized by a planktonic life cycle, belonging to the species *Bythotrephes cederstroemi* (Leydig, 1860) and *Daphnia* spp., are considered intermediate hosts for the genus *Proteocephalus* worldwide [20,21,22]. A morphological evaluation of cestodes belonging to the genus *Proteocephalus* has been performed by some authors. Scholz and colleagues [23] morphologically described cestodes identified as *Proteocephalus tetrastomus* (Rudolphi, 1810) isolated from larval rainbow smelt (*Osmerus mordax*; Mitchill, 1814) sampled in North America. Considering the importance of *A. fallax lacustris* for the Lake Garda environment and that this is one of the most-appreciated species in this area, which has been included since 2008 in the International Union for Conservation of Nature (IUCN) Red List as Least Concern [24], the aim of the present study is to describe the presence of cestodes belonging to the genus *Proteocephalus* in *A. fallax lacustris* caught in Lake Garda.

## 2. Materials and Methods

### 2.1. Sample Collection

Sixty-six specimens were collected during official routine veterinary activities at the Milan fish market (Milan, Lombardy, Italy). All inspected teleosts were caught from Lake Garda using gillnets. After external examination, all fish were opened to evaluate any pathological alterations in celomic organs and for parasitological examination with a stereo microscope (SteREO Discovery.V12 Zeiss, Jena, Germany). At the same time, gill rackers were counted to confirm the fish subspecies. Teleost specimen measurements of BW and TL were taken for each specimen, and the mean weight and mean length (ML) ± standard deviation were calculated and recorded. All parasites found were first rinsed three times using distilled water and then stored in 70% ethanol and transferred to the laboratory of Parasitology and Parasitic Diseases, of the Department of Veterinary Sciences, for morphological identification, and to the Department of Chemical, Biological, Pharmaceutical and Environmental Sciences, University of Messina, for molecular evaluation.

### 2.2. Morphological Evaluation

Some parasites, opportunely selected according to preservation status, were stained by Semichon’s carmine red technique as described by Cable [25], properly modified according to De Benedetto et al. [26]. Briefly, scolex, intermediate and terminal parasite sections were stained in acetocarmine red solution for 12 h, bleached in chloride acid–alcohol solution (1%), washed three times in distilled water to remove any excess of hydrochloric acid, dehydrated in increasing alcoholic solutions for 5 min each (70°, 80°, 90°, 95°, 100°), clarified in clove essential oil (BioSigma, Venice, Italy) for 1 h, and then mounted with Canada balsam. Parasite morphological identification was carried out under an optic microscope (Axioskop 2 plus Zeiss, Jena, Germany) following the keys suggested by Alexander [27], de Chambrier [19] and Scholz [28,29]. All images were acquired with the aid of a digital camera (Axiocam Mrc Zeiss, Jena, Germany) supported by a digital image acquisition system (Axiovision Zeiss, Jena, Germany). Epidemiological indices of prevalence (P%), mean abundance (MA) and mean intensity (MI) were assessed according to Bush et al. [30].

### 2.3. Molecular Analysis 

Parasites were identified also by a molecular approach using DNA extraction (6 specimens) for subsequent PCR amplification and sequence analysis of two different molecular markers, the *large subunit ribosomal RNA (LSU rRNA*) and the *internal transcribed spacer* (*ITS2* region).

#### 2.3.1. DNA Extraction from Parasites

Total DNA was extracted from parasite specimens using the DNeasy Blood & Tissue Kit (QIAGEN, Hilden, Germania) according to manufacturer’s instructions. DNA samples were verified by UV absorbance measurements at 260 and 280 nm (NanoDrop 2000, Thermo Scientific, Wilmington, MA, USA) for quantity, purity, and integrity.

#### 2.3.2. Polymerase Chain Reaction and Sequence Analysis

Genomic DNA was used to amplify the two molecular loci by polymerase chain reactions (PCR) using Taq DNA Polymerase Recombinant kit (Invitrogen, Carlsbad, CA, USA) and the following primer sets 5′AGTCGGGTTGTTTGAGAATG3′ and 5′CGTGTTTCAAGACGGGTC3′ for *LSU rRNA* [31] and 5′ATAGGTGTGTTGTATACGTTGATTGG3′ and 5′AAGCATCGTAATAGCAGC3′ for the *ITS2* region. PCR reactions (50 μL total volume) were performed in an Ep-Gradient Mastercycler (Eppendorf, Hamburg, Germany) using the following cycling parameters for *LSU rRNA*: 94 °C for 30 s, 35 cycles of 94 °C for 30 s, 58 °C for 30 s and 72 °C for 1 min, with a final step of 72 °C for 10 min. For *ITS2*, the following amplification profile was set: 95 °C for 3 min, 40 cycles of 95 °C for 25 s, 48 °C for 25 s and 72 °C for 30 s, with a final extension of 72 °C for 5 min. The PCR products were resolved by 1% agarose gel electrophoresis and amplicons of the expected size were purified using the E.Z.N.A. Gel Extraction Kit (OMEGA, Omega Bio Tek, Norcross, GA, USA), according to manufacturer’s protocol. DNA sequencing of the purified fragments was performed, using both forward and reverse primers for each gene analyzed using the Applied Biosystems 3730 DNA Analyzer (Thermo Fisher Scientific, Wilmington, MA, USA). The obtained DNA sequences were analyzed by BLASTN similarity search against the NCBI database (http://blast.ncbi.nlm.nih.gov/Blast.cgi, accessed on 11 July 2023) and submitted to GenBank database (https://submit.ncbi.nlm.nih.gov/, accessed on 29 July 2023). Phylogenetic relationships were evaluated for the ITS2 marker using several related nucleotide sequences from different species of the *Proteocephalus* genus available in NCBI GenBank. The analysis was performed using MEGA X [32] and maximum likelihood tree, which was constructed selecting the GTR + G + I nucleotide substitution model with the bootstrap method (1000 replications).

## 3. Results

**Figure 1:**
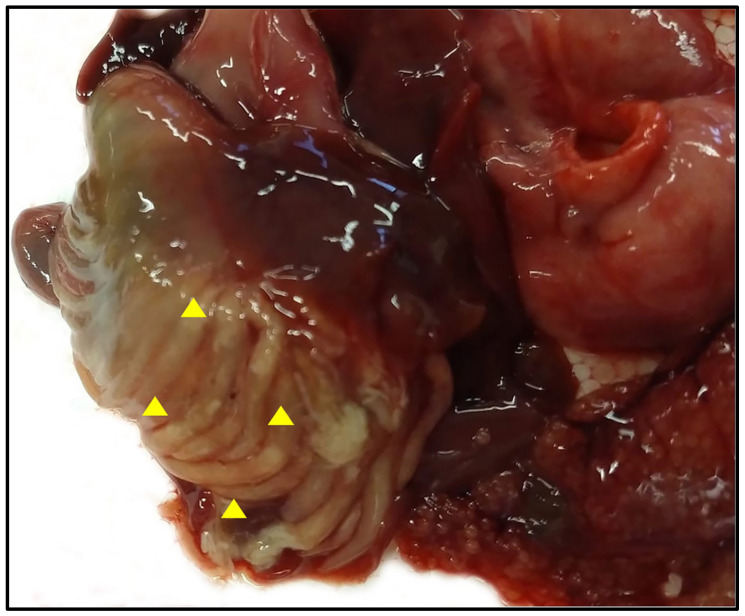
Macroscopic detection of *Proteocephalus longicollis* within the *Alosa fallax lacustris* pyloric caeca (arrowhead).

Gill racker analysis confirmed that all specimens belonged to *Alosa fallax lacustris.* Specimens showed a mean weight of 102 ± 21 g and an ML of 24.4 ± 1.4 cm. Eight out of sixty-six examined teleosts, all investigated during the month of May 2022, were positive for the presence of adult cestodes within the pyloric caeca (P = 12.1%, MA = 1.6, MI = 13.4); some of the positive pyloric caeca appeared to be filled with tapeworms (Figure 1).

No other parasites were macroscopically found in the stomach, intestine and other celomic organs. Morphological features of the examined parasites, considering the scolex, intermediate and terminal sections (proglottids), allowed for us to identify them as belonging to the genus *Proteocephalus*. Morphometric measurements of ML and mean width (MW) ± standard deviation were taken on 6 specimens, which were opportunely selected according to sample preservation, and are reported in micrometres (μm) below.

### 3.1. Morphological Description of Proteocephalus longicollis

**Figure 2:**
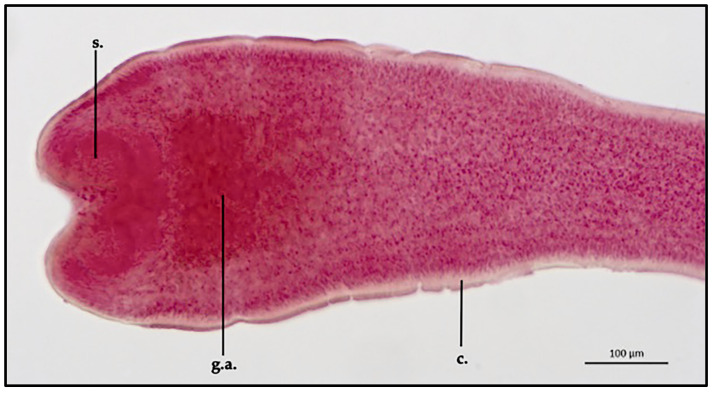
*Proteocephalus longicollis* collected from *Alosa fallax lacustris* after Semichon’s carmine red staining. Scolex, 10x (s., sucker; g.a., gland aggregation, c. cuticle).

*Proteocephalus longicollis* ML and MW (measurement taken on two specimens and reported in mm) were 144.5 ± 2 mm and 2 ± 0.2 mm, respectively. External cuticle appeared non-spinous for the entire parasite body. Examined scolexes appeared rounded to spherical in shape (dorsoventral view). Scolex ML and MW were 536.5 ± 2.2 μm and 298.8 ± 1.1 μm, respectively. In the apical area of the scolex, four small shallow and cup-shaped suckers were present (MW 56.2 ± 0.25 μm); no hooks were observed on the sucker margin and no structure representing a vestigial fifth sucker was seen in the apical area of the scolex. Between the scolex and the neck, numerous gland cells concentrated in the apical regions were observed directly behind the suckers; the glandular area extension had an ML of 208.9 ± 0.8 μm and an MW of 186.9 ± 0.7 μm (Figure 2).

**Figure 3:**
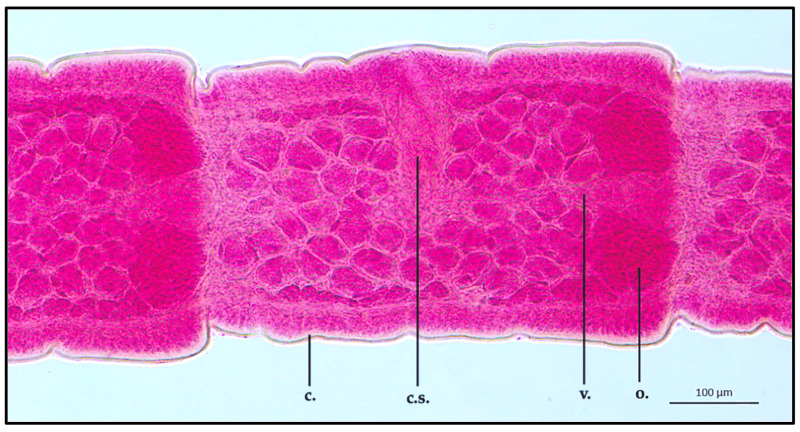
*Proteocephalus longicollis* collected from *Alosa fallax lacustris* after Semichon’s carmine red staining. Immature proglottid, 10x (c., cuticle; c.s., cirrus sac; v., vagina; o., ovary).

Between the scolex and the strobila, a long, thin neck was observed (ML 35,810.2 ± 627.2 μm; MW 126.5 ± 0.9 μm). Directly behind the neck, an immature segment showed an ML of 815.9 ± 0.9 μm and an MW of 323.3 ± 1.5 μm. The excretory system, localized in the mean area of the proglottid, appeared to be alternated in position every three proglottids. Regarding the organs present in the segment, the cirrus sac had an ML and MW of 146.8 ± 1.3 μm and 77.9 ± 0.8 μm, respectively. The vagina appeared to be thin in shape, localized in the medial part of the segment, joining the cirrus, showing an ML of 398.6 ± 1.4 μm. The ovary was observed in the posterior end of the segment, appearing leaf-like in shape (ML 202.4 ± 1.8 μm; MW 110.6 ± 1.2 μm). Due to the immature stage of the examined strobila section, both uterus and testis were not clearly developed (Figure 3).

**Figure 4:**
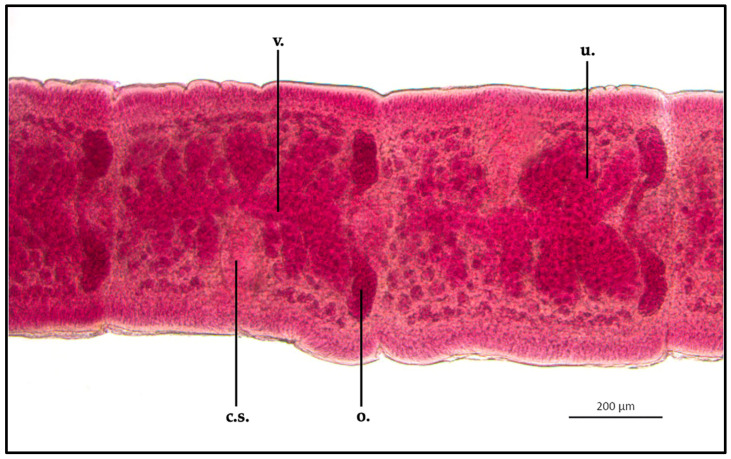
*Proteocephalus longicollis* collected from *Alosa fallax lacustris* after Semichon’s carmine red staining. Mature proglottid, 5x (c.s., cirrus sac; o., ovary; u., uterus; v., vagina).

*Proteocephalus longicollis* mature proglottids appeared clearly different in shape than the immature specimen; ML and MW were 638.8 ± 1.1 μm and 600.3 ± 1.1 μm, respectively. The cirrus sac appeared elongated in shape: ML and MW were 241.9 ± 1 μm and 121.8 ± 0.9 μm, respectively. Uterus appeared split into from 6 to 8 lateral folds from the middle of the organ. The uterus ML was 497.6 ± 0.9 μm, while MW was 373.8 ± 1 μm. The vagina (ML 339.6 ± 1.3 μm) merged with the cirrus sac through the vaginal canal, in the middle of the proglottid, within the uterus body. Testis (MW 19.7 ± 0.8 μm) appeared to be localized in a single layer between the uterus and the external cuticle, which were in the same area, in both sides of the parasite. Some eggs were present in the terminal part of the uterus, close to the cirrus sac (MW 21.8 ± 0.3 μm) (Figure 4).

**Figure 5:**
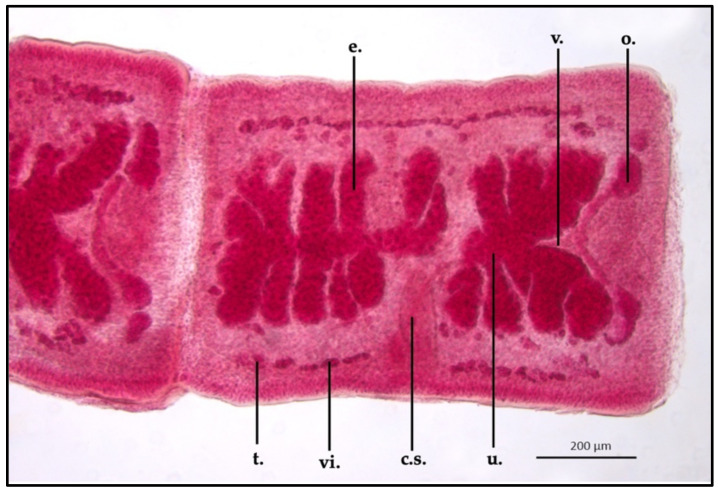
*Proteocephalus longicollis* collected from *Alosa fallax lacustris* after Semichon’s carmine red staining. Mature proglottid, 5x (t., testis; vi., vitellaria; c.s., cirrus sac; u., uterus; o., ovary; v., vagina; e., eggs).

Gravid terminal proglottids appeared more long than they were wide in comparison with the mature section, showing an ML and MW of 932.6 ± 0.8 μm and 658.1 ± 1.3 μm, respectively. The cirrus sac appeared thicker in size than the other development stages: ML was 263.5 ± 1.1 μm and MW 70.3 ± 0.9 μm. The uterus, in this area of the strobila, was the biggest described, reaching an ML and MW of 798.8 ± 1.5 μm and 335 ± 1.5 μm, respectively. In this portion, the uterine folds were closely packed together, filling almost the entire proglottid body. The vagina (ML 469.6 ± 1.1 μm) reached the cirrus sac dorsally and anteriorly, crossing this structure in the ventral portion; in the posterior area, close to the ovary, the vagina appeared wider than the other portions (MW 21.4 ± 0.6 μm). The club-shaped ovary was flattened, appearing larger than the previously described developing stages (ML 395.2 ± 0.9 μm; MW 42.3 ± 0.6 μm). Vitellaria and testis were more marked than the other sections, remaining superimposable in some areas; the testis size was bigger than the other sections, showing an MW of 19.8 ± 0.8 μm. The uterus was filled with round-shaped eggs, which were uniform in size, showing an MW of 23.6 ± 0.1 μm (Figure 5).

### 3.2. Molecular Identification of Proteocephalus longicollis

All the isolates showed positive amplification for both *LSU rRNA* and *ITS2*. The obtained nucleotide sequences were identical among different replicates, and representative sequences of *LSU rRNA* (799 bp) and *ITS2* (211 bp) were deposited in GenBank with accession numbers OR359751 and OR351140, respectively. Sequence analysis and similarity search by BLASTN algorithm for both molecular markers provided similarities with all sequences from different species of the genus *Proteocephalus*, thus confirming that the parasites belong to this genus. In particular, the *ITS2* region was the most suitable molecular marker for species identification with our sequences, sharing 98.5% of their identity with *P. longicollis* (GenBank accession n. AY551166.1), with an E-value 2e-88, query cover 94%.

To evaluate the possibility that the parasites belonged to the species *Proteocephalus fallax*, previously considered a synonym of *Proteocephalus longicollis*, a blast search of the ITS2 sequences was performed against the whole genome shotgun sequence from *Proteocephalus fallax*. The results showed four nucleotide substitutions that were not present in the relative sequences of *Proteocephalus longicollis* in all analyzed individuals.

**Figure 6:**
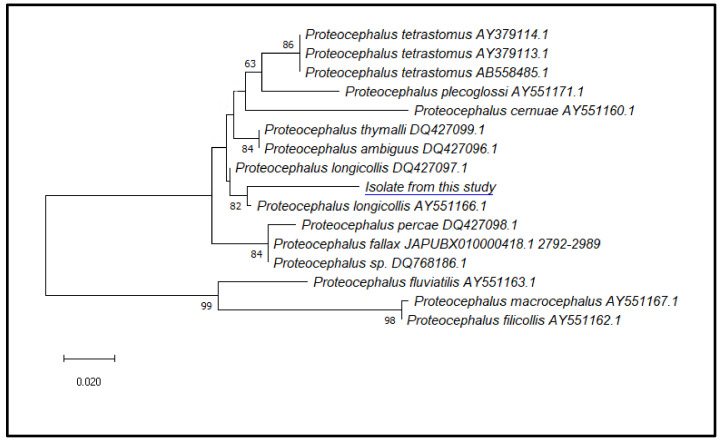
Phylogenetic relationships among the isolates of the present study (*Proteocephalus longicollis*, OR351140) and other species of *Proteocephalus*, as inferred from *ITS2* sequences analysed by maximum-likelihood. Only bootstrap values above 60 are shown. GenBank accession numbers are indicated after the species names (The blue underline represents our isolate).

Phylogenetic analyses of our sequence with the homologous *ITS*2 sequences from different species of the *Proteocephalus* genus previously deposited in GenBank showed that the *ITS2* marker was useful in the species identification, as the sequence from our isolates was in the same clade with *Proteocephalus longicollis* (AY551166.1 and DQ427097.1), supported by a value of 82 at the node and in a separate branch with respect to all the *ITS2* sequences from the other species of *Proteocephalus* retrieved from GenBank (Figure 6).

## 4. Discussion

The present study represents the first report of *Proteocephalus longicollis* isolated from *A. fallax lacustris*, the only species belonging to the family Clupeidae [1] present in Italian deep lakes, adding some information on the parasite fauna of this important freshwater species, which is very appreciated by consumers, and included in the IUCN Red List. In this regard, no data have been reported on the stabilization of *A. fallax lacustris* in Lake Garda. Probably, as described for other areas, this event is related to the adaptation of this species to the characteristics of deep-water Italian lakes, considering possible environment changes at the base of this adaptation, as described by Aprahamian et al. [4]. The number of all *A. fallax lacustris* gill rakers examined in the present study (from 51 to 58) allowed for the identification of all specimens at the species level, confirming the data reported at the beginning by Quignard and Douchement [9], and then by Froese and Pauly [7]. Considering the studies previously published in Italy on *A. fallax lacustris* parasite fauna [15,16], our study represents the first description of parasite infection in this species. Tapeworms of the genus *Proteocephalus* are parasites reported in fish, amphibians and reptiles [33]. Among the fish species, authors reported the presence of parasites belonging to the genus *Proteocephalus* from brook stickleback, *Culaea inconstans* (Kirtland, 1840), sampled in the Neartic Region [29], *Perca fluviatilis* (Linneus 1758) [34] and in coregonid and salmonid fish from North America [35]. In accordance with these data, our study reports the occurrence of *Proteocephalus* sp. in a Clupeidae species from a Southern European Lake for the first time. Alexander [27] described *Proteocephalus salmonidicola* n. sp. in rainbow trout (*Oncorhynchus mykiss*; Walbaum, 1792) sampled from Green Lakes, Deschutes National Forest, United States. In this study, a detailed description of the morphological characteristics has been reported. Considering parasite total length, as well as the characteristics of the other sections evaluated as immature, mature and gravid proglottids, we can confirm the almost total similarity of different species belonging to the genus *Proteocephalus.* Moreover, as reported by Alexander [27], the fifth sucker has not been described, nor any other vestigial structure suggesting an accessory sucker. These results were undoubtedly confirmed by our description. *Proteocephalus longicollis* is considered a common tapeworm found worldwide. Numerous species belonging to the genus *Proteocephalus* were first described from various genera of freshwater fish, in which Scholz and Hanzelová [36] identified the species according to the proglottid total length and width, as well as the number of testes. However, these differences were later considered peculiar, host-related variations due to their species-specific adaptation and characteristics [28,37]. These include the similar scolex shape and positions of the suckers, the shape and size of the vestigial apical sucker, and the large cirrus sac, characterized by a similar size and the specific morphology of the vaginal sphincter [35]. Indeed, according to our results, considering the shape uniformity between *P. longicolllis* described in the present study and the previously reported morphological features, the possible variability, such as length and width of the scolex and proglottids, could be attributable to host-related parasite adaptation. The main morphological difference found between the *P. longicollis* described here and the *P. salmonidicola* n. sp. description reported by Alezander [27] regards the size of the testicles, which are considerably smaller than those described in *P. salmonidicola* n. sp. Morphological characteristics of *Proteocephalus longicollis* have not been sufficiently described and there are difficulties in the identification of specific taxa [38,39,40,41]; in fact, as reported for other species belonging to the genus *Protecephalus*, few characteristics allowed for the parasite to be identified at the species level. In our study, some morphological features have been added. Moreover, a molecular confirmation of the species, based on the *ITS2* sequence, confirms the reported data. Our understanding of the *Proteocephalus*-aggregate was recently debated by Brabec et al. [42], who resolved the diversity of this taxonomically complex group through a novel approach involving high-throughput sequencing (HTS) data-mining. In our study, we questioned the proposed resurrection of the species *Proteocephalus fallax*, previously considered a synonym of *P. longicollis*, by the evaluation of the whole-genome shotgun data and blast results, together with the phylogenetic analysis, supported the identification of *Proteocephalus longicollis* for parasites of *Alosa fallax lacustris*. The scolex morphology is considered one of the most important characteristics used for the identification of cestodes belonging to the genus *Proteocephalus* [33,43]. In agreement with the morphological description reported by Scholz [37], our data, considering the structures present in the scolex and mainly considering the size, confirm the general description of this genus, adding some information about the gland aggregate described in both the scolex and neck region; moreover, the presence of the apical sucker, not found during our evaluation, allowed for us to distinguish the parasite *P. longicollis* described here from the other, previously described, species belonging to the genus *Proteocephalus*. Scholz and colleagues [41] reported the morphological description of *P. tetrastomus* isolated from rainbow smelt. The data reported in that study, compared to our results, could describe some differences between the two species; moreover, the significant reduction in the apical sucker allowed for us to consider this character as a possible evolutionary adaptation of *P. longicollis*. According to Anikieva [20], intermediate stages of *Proteocephalus percae* (Müller, 1780) are characterized by the presence of three pairs of hooks. In our study, as well as in other studies on the genus *Proteocephalus*, no hooks were found; this is probably a typical characteristic of *P. percae*, even if we can speculate on the presence of hooks just during the developing stages. Another significant aspect is represented by the proteocephalidean tapeworm life cycle. Although copepods have usually been considere intermediate hosts of parasites belonging to the genus *Proteocephalus*, these data regarded only experimental trials; therefore, as reported by Scholz [40], copepods can be considered an accidental host for this parasite genus, in which *Proteocephalus* sp. are totally unable to complete their life cycle. Moreover, as reported by Berg and Grimaldi [14], the *A. fallax lacustris* diet is based mainly on crustaceans such as *Daphnia hyalina*, and, for this reason, we can speculate on the role of *D. hyalina* as a possible intermediate host for *P. longicollis*. In addition, a survey conducted by Hanzelová and Gerdeaux [44], including 178 *Coregonus lavaretus* (Linnaeus, 1758) from Lake Annecy (France), reported a high prevalence (up to 90%) of *P. longicollis* infection in copepods throughout the year, recording the highest prevalence in June, while other data showed a complete countertrend, with just 9% of parasites reaching maturity in adult *C. lavaretus*. In our case, the highest prevalence in *A. fallax lacustris* adult specimens was recorded in May; considering the slight difference in lake temperature between May and June, we can consider the possibility of *P. longicollis* infection in adult fish to be partially superimposable with the previously reported results in intermediate hosts.

## 5. Conclusions

Based on the results obtained in the present study, for the first time, it was possible to describe the presence of *Proteocephalus longicollis* in *Alosa fallax lacustris*, the only Clupeidae inhabiting Lake Garda, from a morphological and molecular point of view. Furthermore, from the public health perspective, *P. longicollis*, which is not considered a zoonotic agent, does not represent a risk of parasitic fish-borne zoonosis for consumers of this appreciated teleost from Italian inland waters.

## Data Availability

The data presented in this study are available on request from the corresponding author.
